# Daytime Exposure to Blue Light Alters Cardiovascular Circadian Rhythms, Electrolyte Excretion and Melatonin Production

**DOI:** 10.3390/pathophysiology29010011

**Published:** 2022-03-14

**Authors:** Anna A. Bryk, Mikhail L. Blagonravov, Vyacheslav A. Goryachev, Sergey M. Chibisov, Madina M. Azova, Sergey P. Syatkin

**Affiliations:** 1 V.A. Frolov Department of General Pathology and Pathological Physiology, Institute of Medicine, Peoples’ Friendship University of Russia (RUDN University), 6 Miklukho-Maklaya Street, 117198 Moscow, Russia; blagonravov-ml@rudn.ru (M.L.B.); goryachev-va@rudn.ru (V.A.G.); chibisov-sm@rudn.ru (S.M.C.); syatkin-sp@rudn.ru (S.P.S.); 2 Department of Biology and General Genetics, Institute of Medicine, Peoples’ Friendship University of Russia (RUDN University), 6 Miklukho-Maklaya Street, 117198 Moscow, Russia; azova-mm@rudn.ru

**Keywords:** monochromatic light, blood pressure, heart rate, hypertension, circadian rhythm

## Abstract

Artificial light is characterized by certain features of its impact on the body in terms of its spectral distribution of power, duration of exposure and intensity. Short waves, perceived as blue light, are the strongest synchronizing agent for the circadian system. In the present work, we investigated the features of the circadian rhythms of blood pressure (BP), heart rate (HR), the excretion of electrolytes and the secretion of melatonin in normotensive (Wistar–Kyoto) and hypertensive (SHR) rats under the action of monochromatic blue light in the daytime period. It was found that the exposure of Wistar–Kyoto rats to monochromatic blue light was accompanied by a significant decrease in nighttime and 24 h systolic BP. The most remarkable changes are characteristic of the HR in SHR rats under monochromatic light. A significant decrease in HR in each time period was found, but the predominance of nighttime over daytime values remained in SHR animals. There was also a significant increase in the mesor of the HR in SHR rats. Additionally, the amplitude of diastolic BP and HR, as well as the range of oscillations in HR, were significantly increased compared with the standard light pattern. In contrast to SHR rats, the regulation of the circadian rhythms in Wistar–Kyoto rats was more flexible and presented more changes, which may be aimed at the adaptation of the body to environmental conditions. For Wistar–Kyoto rats, an increase in the level of excreted electrolytes was observed under the action of monochromatic light, but no similar changes were found in SHR rats. For Wistar–Kyoto rats, a significant decrease in the urine concentration of aMT6s in the daytime and nighttime periods is characteristic, which results in the loss of the circadian rhythm. In SHR rats, there was a significant decrease in the nighttime content of aMT6s in the urine, while the daytime concentration, on the contrary, increased. The obtained data demonstrate that prolonged exposure to monochromatic blue light in the daytime period affects the circadian structure of the rhythms of the cardiovascular system, the rhythm of electrolyte excretion and the production of epiphyseal melatonin in wild-type and hypertensive animals. In SHR rats, the rhythms of BP and HR exhibit a more rigid pattern.

## 1. Introduction

Artificial light is an integral component of modern life. It provides illumination when natural light is not available and supports useful activity when darkness falls. People are increasingly exposed to artificial light, which differs from natural light in terms of its spectral power distribution, exposure time and duration, as well as its intensity. Excessive exposure of the body to artificial light at night, known as “light pollution”, is becoming more common in urban environments around the world every year [1,2]. It has been shown in a number of studies that the unrestricted use of electronic devices in the evening led to the self-selection of a later time for sleep, which was certainly associated with the suppression of melatonin, which contributes to better sleep. Additionally, there was also a decrease in evening sleepiness, a deceleration of the circadian rhythm, a later onset of sleep and increased morning sleepiness compared to evenings with the unrestricted use of printed materials only. On the whole, the biological effects of the use of LEDs just before bed are detrimental to performance and health [3,4,5].

Short waves, perceived as blue, are the strongest synchronizing agent for the circadian system, which maintains the internal synchronization of most biological and physiological rhythms. This effect is predominantly realized by inducing the production of epiphyseal serotonin; however, it has a positive and useful character for the functions of the CNS if it only occurs in the morning and early afternoon hours. Despite the presence of a certain amount of research indicating the negative effects of monochromatic light in the evening and at night on the functions of the CNS, there is still very little data describing the regulation of the circadian rhythms of the cardiovascular system, particularly in essential hypertension. The circadian pacemaker—the suprachiasmatic nuclei (SCN)—of the hypothalamus is synchronized by the surrounding light–dark cycles with the help of photoreceptors of the hypothalamic tract. The SCN subsequently synchronize the peripheral “clocks” with each other and the circadian system with geophysical time [6,7,8].

The circadian rhythm is important for optimal body function, and circadian disturbances in sleep–wake cycles or the chronic desynchronization of biological rhythms may even lead to mental and neurodegenerative disorders [9]. Daytime exposure to blue light is important for suppressing the secretion of melatonin—a hormone produced by the pineal gland, which plays a critical role in the regulation of the circadian rhythm. While exposure to blue light is necessary for maintaining proper performance, alertness and cognitive function during the day hours, chronic exposure to low-intensity blue light before bed and at night can have serious consequences for sleep quality, circadian phase and cycle duration [10,11]. Changes in the structure of biological rhythms resulting from the effect of certain environmental factors on the body are associated with the pathogenesis of a number of modern diseases, in particular, essential hypertension [12].

Blood pressure (BP) is regulated by a complex of neurogenic and humoral mechanisms, which largely depend on the state of their chronostructures. The kidneys play a key role in BP control. It is well-known that perfusion pressure in the renal arteries determines the mechanisms of water excretion and the activity of the renin–angiotensin–aldosteron system (RAAS). In the case of kidney pathology, electrolyte metabolism is disordered, the RAAS is activated, and the afferent renal sympathetic activity increases, which contribute to the development and maintenance of hypertension [13,14].

At present, there is no clear understanding of the effects of excessive exposure to visible light, with a predominance of the short-wavelength part of the spectrum, on the chronostructure of the cardiovascular and excretory systems in essential hypertension.

Under natural conditions, the short-wavelength part of the visible spectrum is gradually declined towards the evening and nighttime. However, in the modern world, the duration of exposure to the blue part of the spectrum is significantly extended in time, mostly due to the use of artificial light and electronic LED devices. In our experiment, we simulated the conditions of prolonged action of blue lighting by the animal exposure to isolate the short-wavelength part of the spectrum for 12-h periods every day.

In the present work, we investigated the features of the circadian rhythms of BP, the heart rate (HR), the excretion of electrolytes and the secretion of melatonin in normotensive (Wistar–Kyoto) and hypertensive (SHR) rats under the action of monochromatic blue light in the daytime period.

## 2. Materials and Methods

### 2.1. Animals and Housing

A total of 20 male rats, including 10 SHR (spontaneously hypertensive rats) and 10 Wistar–Kyoto rats (controls), were used in the experiment. The animals were obtained from the Nursery for Laboratory Animals “Pushchino” (branch of the Shemyakin–Ovchinnikov Institute of Bioorganic Chemistry of the Russian Academy of Sciences). By the beginning of the experiment, the animals were 34–36 weeks old. Before beginning the research, all animals were acclimatized for 2 weeks in the laboratory where the experiment was carried out. During the experiment, each animal was kept in an individual cage in artificial light under a free-motion regime and with free access to water and food. The animals were consistently fed at the same time—19:00 h. The room was kept at a constant temperature—+23 °C. The experiment was carried out in accordance with the European Convention for the Protection of Vertebrate Animals used for Experimental and Other Scientific Purposes (Strasbourg, 18 March 1986) and was also approved by the Ethical Committee of the RUDN Institute of Medicine.

### 2.2. Experimental Design

The experiment was performed in 2 separate series of 10 animals. In the first series (SHR, *n* = 5; Wistar–Kyoto, *n* = 5) of the telemetric monitoring of BP, biopotentials of the heart were performed under a standard artificial light–dark pattern, with a ratio of light and dark phases of 12:12 h (light phase—07:00 h–19:00 h, lighting of 350 lux at the level of the animals’ eyes; dark phase—19:00 h–07:00 h, lower than 0.5 lux). For the first 7 days, ordinary white light was used during the light phase (control). For the next 7 days, the animals were exposed to monochromatic light with a wavelength of 520 nm in the phase. The assessment of the corresponding indicators was carried out on the 7th day of each period. In the second series, the animals were divided into groups in the same way as in the first series. The excretion of electrolytes (Na^+^, K^+^, Ca^2+^ and Mg^2+^) in the urine was assessed using capillary electrophoresis. In the same samples of urine, the concentration of 6-sulfatoxymelatonin (aMT6s) was evaluated using an ELISA.

### 2.3. Monitoring of BP and ECG

The continuous 24 h registration of BP and ECG in standard lead II was carried out with a telemetric monitoring technique using the radio telemetry system DSi (New Brighton, MN, USA). For this purpose, DSi HD-S11 radio transmitters were surgically implanted, under general anesthesia, into the animals (Zoletil, Virbac(Carros, France), EC). Radio transmitters are devices that monitor BP, biopotentials of the heart, body temperature and activity, and transmit data as a radio signal to special receivers placed near animals’ cages. BP was monitored using a catheter installed in the lumen of the abdominal aorta and fixed with a tissue hemostatic adhesive. For ECG monitoring, electrodes were fixed under the chest muscles in the projection of the electrical axis of the heart. Recording of all the mentioned parameters was started 10 days after the implantation of transmitters.

The obtained data were processed using the DataquestA.R.T. (St. Paul, MN, USA, Version 4.2 Gold) and ChronosFit (Heidelberg, Germany, Version 1.05) software. In rats of the control and experimental groups, the following parameters were determined for 2-min periods with intervals of 15 min for 24 h periods: systolic blood pressure (SBP), diastolic blood pressure (DPB) and heart rate (HR). Furthermore, the obtained data were evaluated by the methods of linear and nonlinear rhythm analysis using the ChronosFit program [15].

For all the indicators, the method of linear analysis was used to determine the daytime average (from 07:00 h to 19:00 h), nighttime average (from 19:00 h to 07:00 h) and daily average (form 07:00 h to 07:00 h of the next day) values. Therefore, the following indicators were assessed: daytime systolic blood pressure (SBPday), daytime diastolic blood pressure (DBPday), daytime heart rate (HRday), nighttime systolic blood pressure (SBPnight), nighttime diastolic blood pressure (DBPnight), nighttime heart rate (HRnight), daily systolic blood pressure (SBP24h), daily diastolic blood pressure (DBP24h) and daily heart rate (HR24h).

Nonlinear analysis is a combination of partial Fourier analysis with stepwise regression. Using nonlinear analysis for SBP, DBP and HR, the following indicators were determined: mesor—the average level of the indicator for a 24 h period; max and min—maximum and minimum values of the corresponding parameter over a 24 h period; range of oscillations—the difference between the maximum and minimum value of an indicator; % rhythm (power of oscillations)—chronobiological index reflecting the proportion of oscillatory processes (the proportion of the indicator values having an oscillatory distribution for a 24 h period); and amplitude—maximum deviation of the corresponding indicator from the mesor for 24 h.

### 2.4. Assessment of Electrolyte Excretion

The capillary electrophoresis (CE) technique was used to measure the levels of Na^+^, K^+^, Ca^2+^ and Mg^2+^ excreted with urine for a 24 h period (07:00 h–07:00 h), the daytime period (07:00 h–19:00 h) and the nighttime period (19:00 h–07:00 h). To collect urine, metabolic cages for rats, AE0906, produced by the production company “Open Science” (Krasnogorsk, Russia), were used. The concentration of electrolytes in urine was determined using the CE system “Kapel-105M”, with the methods and reagents of the company “Lumex” (Saint Petersburg, Russia). The amount of excreted electrolytes was calculated by taking into account the data on the volume of urine in the corresponding samples.

### 2.5. Assessment of Epiphyseal Melatonin Secretion

Changes in the blood concentration of melatonin have a marked circadian rhythm, commonly with higher levels at night. Most circulating melatonin is metabolized in the liver into 6-hydroxymelatonin and then into 6-sulfatoxymelatonin (aMT6s), which is excreted with urine. The concentrations of epiphyseal melatonin and aMT6s in the urine are in direct correlation [16,17,18]. The concentration of aMT6s was measured in urine collected in the daytime (07:00 h–19:00 h) and nighttime (19:00 h–07:00 h) periods using a 6-Sulfatoxymelatonin ELISA (Buhlmann Laboratories AG, Switzerland). For this purpose, the samples of urine collected for CE (item 2.4) were used. An ELISA was performed using a Sunrise absorbance microplate reader (Tecan, Austria).

### 2.6. Statistics

Statistical analysis of the obtained data was carried out using the program “STATISTICA 6.0” (StatSoft, Inc., Tulsa, OK, USA). The mean value and the error of the mean were counted for each of the studied indices. The significance of the differences in the means was checked using the Mann–Whitney U test (the difference in the mean values was taken as significant at *p* ≤ 0.05).

## 3. Results

### 3.1. Telemetric Monitoring of BP and HR

**Figure 1:**
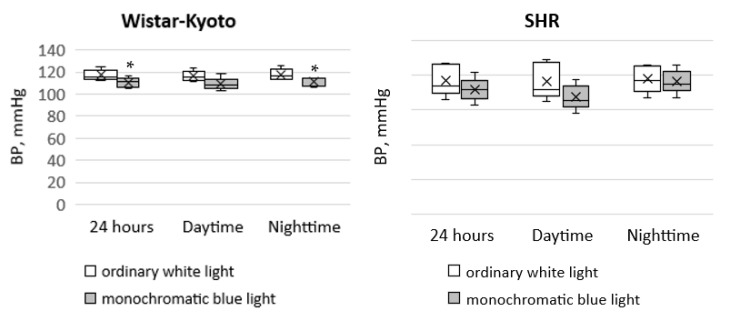
Indicators of the telemetric monitoring of SBP in Wistar–Kyoto and SHR rats under the standard light pattern (12 h:12 h) with ordinary white light and monochromatic blue light. *—*p* ≤ 0.05.

In Wistar–Kyoto rats, the tendency for a decrease in SBP_day_ was noted under the action of monochromatic blue light compared to standard lighting with ordinary blue light. At the same time, there was a significant decrease in SBP_night_ and SBP_24h_. In SHR rats, only a similar tendency was seen; however, there were no significant differences (Figure 1).

**Figure 2:**
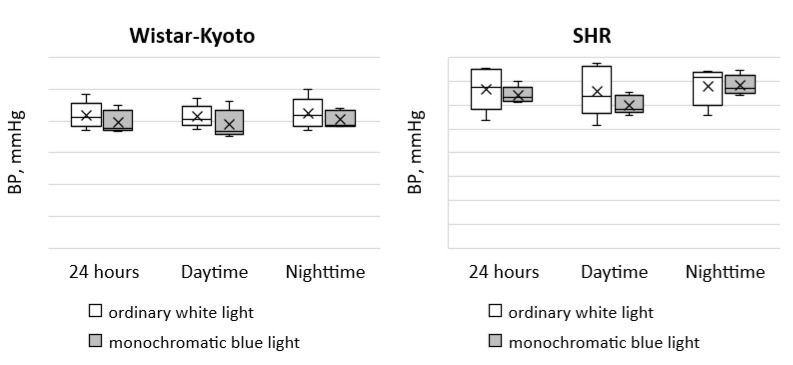
Indicators of the telemetric monitoring of DBP in Wistar–Kyoto and SHR rats under the standard light pattern (12 h:12 h) with ordinary white light and monochromatic blue light.

Moreover, the use of monochromatic light did not induce any changes in DBP in the animals of both genetic strains (Figure 2).

For Wistar–Kyoto and SHR rats, no significant changes were observed between SBP_day_ and SBP_night_ as well as DBP_day_ and DBP_night_ under standard lighting with ordinary white light. At the same time, exposure to monochromatic light did not lead to any changes in the 24 h distribution of BP in Wistar–Kyoto rats, but in SHR rats, the circadian rhythm of DBP was characterized by the predominance of nighttime over daytime values.

**Figure 3:**
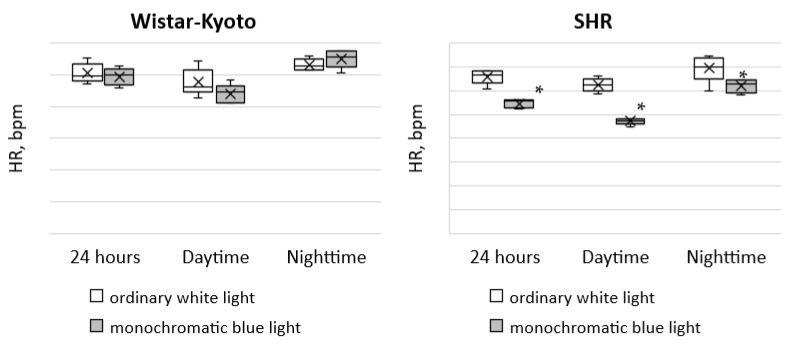
Indicators of the telemetric monitoring of HR in Wistar–Kyoto and SHR rats under the standard light pattern (12 h: 12 h) with ordinary white light and monochromatic blue light. *—*p* ≤ 0.05.

The most considerable changes are characteristic of the HR in SHR rats under monochromatic light. A significant decrease in HR in each time period was found, but the predominance of nighttime over daytime values remained (Figure 3).

It is important that a significant excess of HR_night_ over HR_day_ is typical for Wistar–Kyoto and SHR rats in both ordinary white and monochromatic lighting.

**Figure 4:**
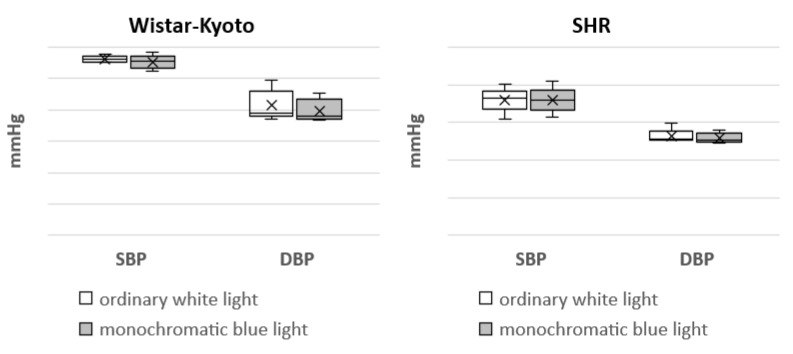
Mesor of SBP and DBP in Wistar–Kyoto and SHR rats under the standard light pattern (12 h:12 h) with ordinary white light and monochromatic blue light.

**Figure 5:**
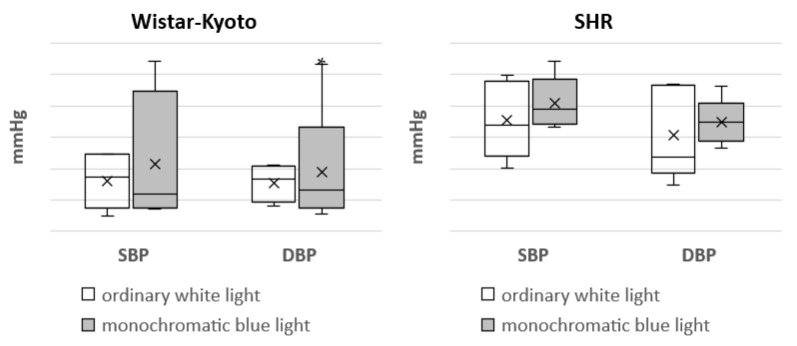
Amplitude of SBP and DBP in Wistar–Kyoto and SHR rats under the standard light pattern (12 h:12 h) with ordinary white light and monochromatic blue light. *—*p* ≤ 0.05.

**Figure 6:**
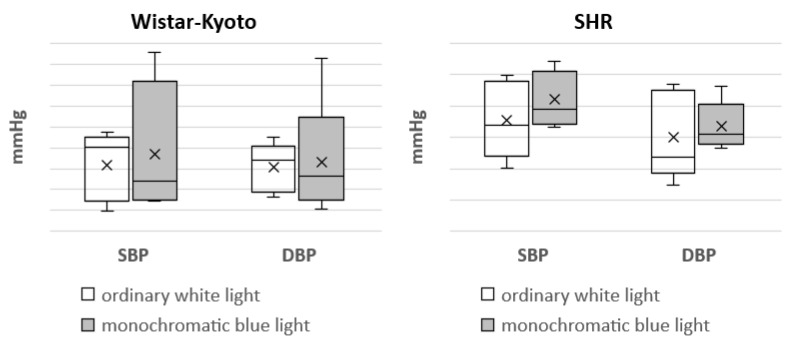
Range of oscillations in SBP and DBP in Wistar–Kyoto and SHR rats under the standard light pattern (12 h:12 h) with ordinary white light and monochromatic blue light.

**Figure 7:**
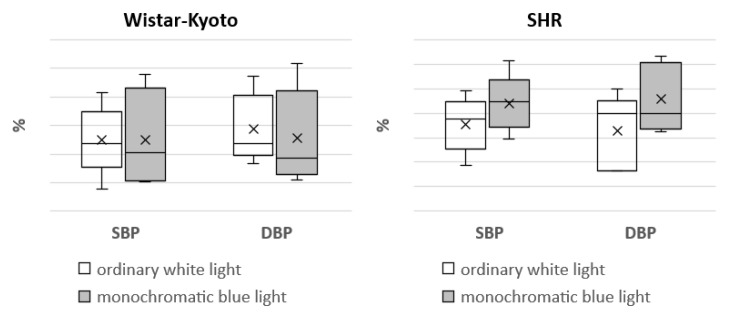
Rhythm power (% of rhythm) of SBP and DBP in Wistar–Kyoto and SHR rats under the standard light pattern (12 h:12 h) with ordinary white light and monochromatic blue light.

**Figure 8:**
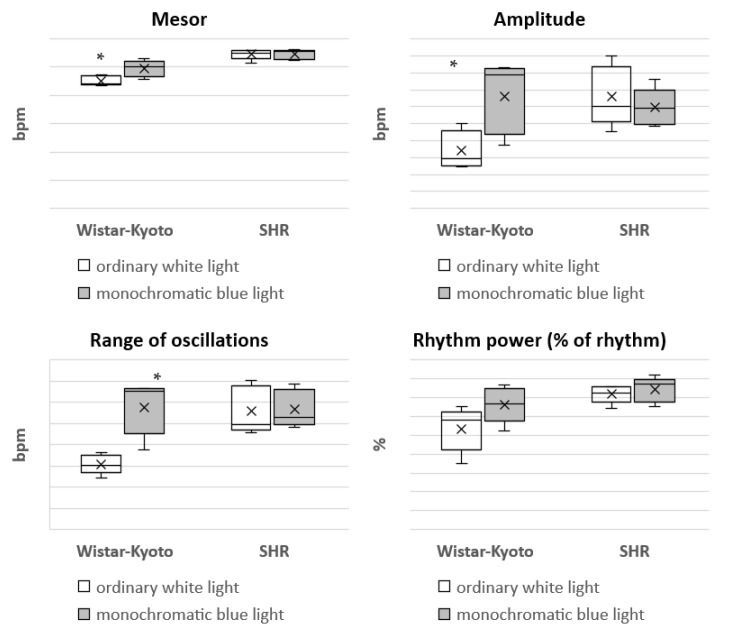
Indicators of 24 h HR profiles obtained on the basis of nonlinear analysis in Wistar–Kyoto and SHR rats under the standard light pattern (12 h:12 h) with ordinary white light and monochromatic blue light. *—*p* ≤ 0.05.

In Wistar–Kyoto rats, the mesor of SBP and DBP did not present any significant changes under monochromatic light in comparison with ordinary white lighting. However, there was a significant increase in the mesor of HR. The amplitude of DBP and HR, as well as the range of oscillations of HR, were significantly increased compared with the standard light pattern. In regard to the same rhythmological indices of BP and HR in SHR rats, no significant differences were found under the action of monochromatic blue light (Figure 4, Figure 5, Figure 6, Figure 7 and Figure 8).

Hence, under the predominance of the blue spectrum in the visible light, the chronostructure of the BP and HR in Wistar–Kyoto rats was more flexible and showed more changes, which are probably aimed at the adaptation of the cardiovascular system to the environmental conditions.

### 3.2. Electrolyte Excretion

In Table 1, the data reflected the amount of electrolytes (Na^+^, K^+^, Ca^2+^ and Mg^2+^) excreted with urine for the 24 h, daytime and nighttime periods under ordinary white light and monochromatic blue light.

In Wistar–Kyoto rats, the circadian rhythm of Na^+^, K^+^ and Mg^2+^ excretion with a predominance of nighttime over daytime values was found under the standard light pattern. For Ca^2+^, only a similar tendency was typical, but the difference was not statistically significant. Under monochromatic light, there was a sharp enhancement of Na^+^ excretion in all the studied periods; the excretion of K^+^ and Mg^2+^ significantly increased in the daytime hours, and the excretion of Ca^2+^ increased for the 24 h and daytime periods. Due to an increase in the daytime excretion of Mg^2+^, its circadian rhythm was also changed: the difference between the daytime and nighttime values disappeared, while a similar increase in the levels of Na^+^ and K^+^ during the day did not induce any changes, and their circadian rhythm of excretion remains unchanged.

In contrast, for SHR rats, no differences between the daytime and nighttime values of electrolyte excretion were characteristic under the standard light pattern (12 h:12 h) with ordinary white light. Thereby, long-term hypertension was accompanied by the disappearance of the circadian pattern in electrolyte excretion. The exposure of the animals to the conditions of blue spectrum predominance did not lead to any significant changes in electrolyte excretion. The only exception was the daytime excretion of K^+^, which was significantly lower than that under the standard light pattern, resulting in the formation of a circadian rhythm in the excretion of this ion (the daytime value was 1.98 mmol, while the nighttime value was 3.99 mmol). A slight decrease in the excretion of all the studied electrolytes may probably be explained by a decrease in diuresis under simulated experimental monochromatic lighting. On average, the amount of excreted urine decreased in the daytime by 1.83 times, and at night by 1.87 times.

### 3.3. Epiphyseal Melatonin Secretion

**Figure 9:**
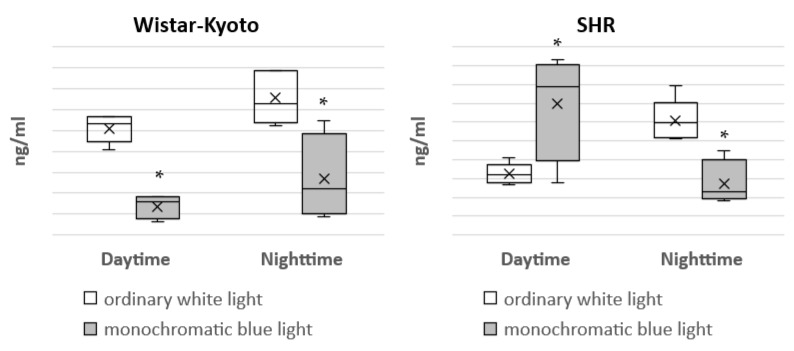
Urinary concentration of aMT6s in Wistar–Kyoto and SHR rats under the standard light pattern (12 h:12 h) with ordinary white light and monochromatic blue light. *—*p* ≤ 0.05.

Under the standard light pattern (12 h:12 h), in Wistar–Kyoto rats, there was a distinct circadian rhythm in the concentration of aMT6s in urine, with a predominance of nighttime over daytime values. Under the action of monochromatic blue light, a significant decrease in the concentration of aMT6s in the daytime and nighttime hours was characteristic, which led to the loss of its circadian rhythm (Figure 9).

In SHR rats, the daytime level of aMT6s in urine was significantly lower in comparison with night hours under the standard light pattern with ordinary white light. However, under the action of monochromatic light, a significant decrease in the content of aMT6s in urine was noted at night, but its daytime concentration, on the contrary, increased. As a result, the difference between the nighttime and daytime levels of this metabolite in urine persisted, but in an inverted form. The daytime value of aMT6s was 34.81 ng/mL, and the nighttime value was 13.64 ng/mL. Apart from the changes associated with the action of monochromatic light, some other features of melatonin metabolism in Wistar–Kyoto and SHR rats were also observed. Under the standard light pattern with ordinary white light, the daytime concentration of aMT6s in urine in SHR rats was significantly lower compared with Wistar–Kyoto rats, but, on the contrary, under monochromatic lighting, it was significantly higher.

## 4. Discussion

In the present experiment, indicators reflecting the circadian rhythms of the cardiovascular system, the excretion of electrolytes and the production of epiphyseal melatonin under exposure to monochromatic blue light were studied. The results of telemetric monitoring showed that the circadian rhythm of HR was typical for both the standard light pattern with ordinary white light and for monochromatic lighting. Circadian rhythms are characteristic of many functions of the cardiovascular system, including HR, BP and molecular as well as gene reactions. In the case of cardiovascular pathology, survival largely depends on the maintenance of normal circadian rhythms [19,20].

Under the action of monochromatic light, a significant decrease in SBP_24h_ and SBP_night_ was seen in Wistar–Kyoto rats. In SHR rats, only a similar tendency was noted; however, there were no statistically significant differences. In the study performed by M. Stern et al. [21], it was shown that exposure to blue lighting for 30 min over 2 days resulted in a significant decrease in SBP compared to the controls. The hemodynamic effect of blue light can be presumably explained by the release of NO into circulating blood [21]. These data were obtained in a group of healthy volunteers with normal BP, and these findings are consistent with what we have observed in normotensive Wistar–Kyoto rats. However, in the SHR animals, we did not find similar changes, which indicates a higher rigidity of the vascular tone control in comparison with Wistar–Kyoto rats. In the present experiment, under the action of monochromatic light, a significant decrease in 24 h and nighttime SBP was seen in Wistar–Kyoto rats. In SHR rats, only a similar tendency was noted; however, there were no significant differences. This is probably due to the fact that, in the case of arterial hypertension, there is a depression in the synthesis of endothelial NO. In general, it can be noted that, in SHR rats, exposure to monochromatic blue light does not significantly affect daytime and nighttime SBP and DBP, but reduces 24 h, daytime and nighttime HR, bringing them closer to the normal values, which is most probably due to an inhibition of the sympathetic activity. These findings allow us to consider the action of blue light as a positive factor for the cardiovascular system, particularly in hypertension.

Earlier studies have shown that resting HR is affected by the day/night cycle and light levels [22,23,24]. It is known that exposure to light of different colors (wavelengths) and intensities affects HR variability through various mechanisms, including the modulation of sympathovagal balance, the renin–angiotensin–aldosterone system, the endocrine system acting through anterior and posterior hypothalamic stimulation, etc. [25]. Various studies have been carried out to identify and quantify the effect of different colors of light on HR variability, but conclusions were indefinite and sometimes even contradictory [26,27,28]. In our study, a significant decrease in HR was found in every time period.

The nonlinear analysis of BP and HR rhythm in SHR rats did not reveal any marked changes in the mesor, range of oscillations, amplitude and power of the rhythm (% rhythm). However, in Wistar–Kyoto rats, an increase in the mesor, amplitude and range of oscillations of HR as well as the amplitude of DBP was noted. Hence, the rhythmic organization of some functions of the cardiovascular system in Wistar–Kyoto rats is characterized by higher flexibility and mobility. It can be suggested that these changes are aimed at expanding the adaptive capacities of the body. At the same time, the absence of rhythmological changes in BP and HR in SHR rats indicated a more stable and rigid character of these functions. The obtained data are consistent with our previous studies, showing similar patterns of the responses under free-run rhythm and the conditions of prolonged daylight duration [29,30].

In regard to the excretion of electrolytes, for Wistar–Kyoto rats kept under the standard light pattern, a pronounced circadian rhythm of Na^+^, K^+^ and Mg^2+^ excretion, with a predominance of nighttime over daytime values, was characteristic. For Ca^2+^, the differences were not significant. Similar findings were described earlier. In particular, in the experiment on male rats, it was shown that the excretory function of the kidneys at night was higher by more than 40% in comparison with the daytime period [31]. The exposure of Wistar–Kyoto rats to the conditions of blue spectrum predominance led to the disappearance of the circadian rhythm of Mg^2+^ excretion, while the rhythms of Na+ and K+ excretion remained unchanged. Apparently, the excretion of these electrolytes is more resistant to the influence of external factors. The action of monochromatic lighting was accompanied by an increase in the level of excretion of all the studied ions. An increase in Na+ excretion was observed in all time periods; the excretion of K^+^ and Mg^2+^ significantly increased in the daytime hours, and Ca^2+^ excretion was also increased in the daytime and 24 h periods. It was also shown that acute sleep deprivation induced natriuresis and osmotic diuresis, leading to excess nocturnal urine production, especially in men [32].

Kidney function is also under the control of the “circadian clock”. It has been shown in rodent models that the dysregulation of the “molecular clock” was associated with the aggravation of the kidney state and hypertension [33]. It has also been demonstrated that the pineal gland of rats controls the ion-excretory function of the kidneys and the circadian rhythm of diuresis. Additionally, exogenous light stimuli, forming the daily rhythm of the kidneys, are mediated through the pineal gland [34]. The light–dark cycle is the main synchronizer of circadian rhythms in the excretion of water and electrolytes. In the absence of a cyclic light pattern, the persistent frequency of food intake is not a sufficiently powerful synchronizer of the circadian rhythms of water and electrolyte excretion [35,36]. It was established that the hypofunction of the rat pineal gland indicated a disorder of the phase structure of the ion-regulating function compared with the chronograms of the intact rats. After an experimental pinealectomy in animals, an increase in the excretion of Na^+^ was observed, while its blood concentration remained elevated around the clock [37]. In our study, a decrease in the content of melatonin was also noted, which may have led to an increase in the excretion of electrolytes. At the same time, in some studies, an association between an enhancement of natriuresis and a decrease in aldosterone levels is suggested. The “circadian clock” has been shown to control Na^+^ reabsorption in the kidneys through the modulation of aldosterone production by the adrenal cortex [38,39,40].

In our study, there was no circadian rhythm in the excretion of electrolytes in SHR animals under the standard light pattern. A long course of hypertension likely leads to a disorder of the “biological clocks” in the kidneys, accompanied by the disappearance of the circadian rhythm of electrolyte excretion; however, we cannot exclude kidney damage due to hypertension as the leading mechanism [41,42]. At the same time, as shown in some other studies, the concentration of plasma sodium obeys the circadian rhythm, which is inversely related to the circadian rhythm of BP. Although plasma sodium rhythms in SHR and Wistar–Kyoto rats are almost identical, the concentration of plasma sodium is significantly higher in SHR rats throughout the 24 h cycle [43]. The exposure of the animals to the conditions of blue spectrum prevalence did not lead to any significant changes in the rate of excreted electrolytes. The observed decrease in diuresis contributed to a slight decrease in the intensity of electrolyte excretion. For K^+^, differences were significant. Thus, we observed liquid and electrolyte retention in SHR rats, which allows us to assume the existence of a pathological system leading to an even more profound disadaptation of the body under new environmental conditions.

Earlier, we also studied the features of electrolyte metabolism in SHR rats at the initial stages of essential hypertension. According to the obtained data, there was an increase in the rate of Na^+^ and Ca^2+^ excretion, which is suggestive of a compensatory reaction of the body. However, over time Na^+^ retention was observed [44].

At present, it is generally accepted that melatonin secretion is suppressed in hypertension. In hypertensive patients, the normal production of melatonin is observed during the day, but in the nighttime hours, it is decreased. A similar pattern is typical for patients suffering from hypertension without a physiological decrease in BP at night. Apart from hypertensive patients, melatonin deficiency was also found in the case of coronary heart disease [45]. The results of the present study showed that the content of aMT6s in the urine of SHR rats was significantly lower during the daytime period (16.27 ± 1.23 ng/mL) compared with Wistar–Kyoto rats (25.5 ± 1.49 ng/mL). However, at the same time, we observed a distinct circadian rhythm of melatonin secretion in the animals of both genetic strains under the standard light pattern. Therefore, we can conclude that long-term essential hypertension does not affect the circadian rhythm of melatonin secretion by the pineal gland. The prolonged exposure of animals to monochromatic lighting with the predominance of the blue spectrum was accompanied by different reactions in SHR and Wistar–Kyoto rats.

The action of the blue light in the daytime (from 07:00 h to 19:00 h) caused a significant decrease in the content of aMT6s in urine in Wistar–Kyoto rats for the 24 h period. Such a significant decrease in the aMT6s level during the daytime and nighttime periods led to the loss of the circadian rhythm in melatonin secretion. Disturbances of the circadian pattern in melatonin production were also found in some of our previous experiments. In particular, an increase in the duration of the light phase of the day to 20 h (the ratio of light/dark phases was 20 h:4 h) induced the complete loss of the circadian rhythm of melatonin secretion in both Wistar–Kyoto and SHR rats [46]. Cajochen et al. [25] showed that the most profound suppression of melatonin secretion was observed under the action of blue light (460 nm). It was also found that blue light inhibits the production of melatonin and worsens sleep, but, on the other hand, during the light phase, it enhances cognitive functions and improves performance [47]. A similar reaction is observed when exposed to artificial light at night. It was proven that melatonin secretion is suppressed in this case [48,49].

The response of SHR rats to prolonged exposure to monochromatic blue light is of the greatest interest. Under this light pattern, an inversion of the circadian rhythm of melatonin secretion was observed, which occurred due to a significant decrease in the aMT6s level at night and to an increase in the daytime period. We have to mention that an increase in the concentration of aMT6s over the day has already been noted in some of our previous work. In SHR rats, the prolongation of the light phase of the day to 20 h (the ratio of light/dark phases was 20 h:4 h) was accompanied by a significant increase in the aMT6s content in urine in the daytime period; under the standard light pattern (12 h:12 h), it was 16.27 ± 1.23 ng/mL, and under the light/dark pattern (20 h:4 h), it was elevated to 27.39 ± 1.13 ng/mL [30]. It can be assumed that an increase in the daytime blood level of melatonin both under the extended light phase of the day and in the case of monochromatic blue light action is associated with an enhancement of serotonin (5-hydroxytryptamine) formation. Such a change in melatonin secretion is probably explained by its phase shift. Changes in the dark/light cycle have both acute and chronic effects on the melatonin rhythm. Even single flashes of bright light can affect the rhythm of melatonin. Moreover, the effects of light, which are not responsible for image formation, are largely dependent on some specific properties, including intensity, duration, time, structure and wavelength [50,51]. Light with certain characteristics can both delay and advance the circadian system. It was noted in some studies that short-wavelength light (with light wavelengths between 435 and 540 nm) is more effective than long-wavelength light in suppressing nighttime melatonin and slowing the melatonin rhythm phase [52,53,54]. Since we did not measure the blood concentration of melatonin but its metabolite in urine, it should also be taken into account that the acrophase of aMT6s is located approximately 2 h after the acrophase of plasma melatonin [55]. Hence, a phase shift of melatonin secretion towards its delay cannot be excluded.

## 5. Conclusions

The obtained data have shown that prolonged exposure to monochromatic blue light in the daytime period does affect not only the circadian structure of rhythms of the cardiovascular system but also the rhythm of electrolyte excretion and the production of epiphyseal melatonin, both under normal and increased BP. It was also found that the rhythms of BP and HR are more rigid to the action of monochromatic light in the case of essential hypertension.

## Figures and Tables

**Table 1:** Indicators of electrolyte excretion in Wistar–Kyoto and SHR rats under the standard light pattern (12 h:12 h) with ordinary white and monochromatic blue light (mmol, M ± m).

Parameter	Ordinary White Light	Monochromatic Blue Light
Wistar-Kyoto rats		
24 h period		
Na^+^	1.70 ± 0.25	19.93 ± 3.07 *
K^+^	4.8 ± 0.99	7.93 ± 1.14
Ca^2+^	0.17 ± 0.02	0.40 ± 0.08 *
Mg^2+^	0.83 ± 0.12	1.07 ± 0.14
Daytime period		
Na^+^	0.16 ± 0.06	7.15 ± 1.39 *
K^+^	1.04 ± 0.22	2.55 ± 0.58 *
Ca^2+^	0.07 ± 0.02	0.23 ± 0.06 *
Mg^2+^	0.22 ± 0.04	0.50 ± 0.10 *
Nighttime period		
Na^+^	1.54 ± 0.28 ♦	12.78 ± 1.95 ♦*
K^+^	3.76 ± 0.84 ♦	5.39 ± 0.58 ♦
Ca^2+^	0.1 ± 0.01	0.16 ± 0.03
Mg^2+^	0.61 ± 0.08 ♦	0.57 ± 0.11
SHR rats		
24 h period		
Na^+^	3.94 ± 1.5	1.63 ± 0.65
K^+^	7.96 ± 2.31	5.97 ± 0.74
Ca^2+^	0.23 ± 0.08	0.19 ± 0.03
Mg^2+^	1.52 ± 0.39	0.88 ± 0.17
Daytime period		
Na^+^	1.07 ± 0.19	1.44 ± 0.39
K^+^	3.92 ± 0.62	1.98 ± 0.27 *
Ca^2+^	0.08 ± 0.02	0.11 ± 0.01
Mg^2+^	0.73 ± 0.12	0.60 ± 0.14
Nighttime period		
Na^+^	2.86 ± 1.34	0.98 ± 0.19
K^+^	4.04 ± 1.76	3.99 ± 0.61 ♦
Ca^2+^	0.15 ± 0.06	0.09 ± 0.02
Mg^2+^	0.79 ± 0.3	0.28 ± 0.06

Note: *p* ≤ 0.05 *—in comparison with the standard light pattern (12 h:12 h) with ordinary white light; ♦—in comparison with the daytime values.

## Data Availability

Data are contained within the article.

## References

[1] Davies T.W., Smyth T. (2018). Why artificial light at night should be a focus for global change research in the 21st century. Glob. Chang. Biol..

[2] Falchi F., Furgoni R., Gallaway T.A., Rybnikova N.A., Portnov B.A., Baugh K., Cinzano P., Elvidge C.D. (2019). Light pollution in USA and Europe: The good, the bad and the ugly. J. Environ. Manag..

[3] Chang A.M., Aeschbach D., Duffy J.F., Czeisler C.A. (2015). Evening use of light-emitting eReaders negatively affects sleep, circadian timing, and next-morning alertness. Proc. Natl. Acad. Sci. USA.

[4] Chinoy E.D., Duffy J.F., Czeisler C.A. (2018). Unrestricted evening use of light-emitting tablet computers delays self-selected bedtime and disrupts circadian timing and alertness. Physiol. Rep..

[5] Smith A.K., Conger J.R., Hedayati B., Kim J.J., Amoozadeh S., Mehta M. (2020). The Effect of a Screen Protector on Blue Light Intensity Emitted from Different Hand-held Devices. Middle East Afr. J. Ophthalmol..

[6] Arushanian E.B., Baturin V.A., Popov A.V. (1988). The suprachiasmatic nucleus of the hypothalamus as a regulator of the circadian system of mammals. Usp. Fiziol. Nauk..

[7] Weaver D.R. (1998). The suprachiasmatic nucleus: A 25-year retrospective. J. Biol. Rhythm..

[8] Herzog E.D., Hermanstyne T., Smyllie N.J., Hastings M.H. (2017). Regulating the Suprachiasmatic Nucleus (SCN) Circadian Clockwork: Interplay between Cell-Autonomous and Circuit-Level Mechanisms. Cold Spring Harb. Perspect. Biol..

[9] Tähkämö L., Partonen T., Pesonen A.K. (2019). Systematic review of light exposure impact on human circadian rhythm. Chronobiol. Int..

[10] Gabel V., Maire M., Reichert C.F., Chellappa S.L., Schmidt C., Hommes V., Viola A.U., Cajochen C. (2013). Effects of artificial dawn and morning blue light on daytime cognitive performance, well-being, cortisol and melatonin levels. Chronobiol. Int..

[11] Wahl S., Engelhardt M., Schaupp P., Lappe C., Ivanov I.V. (2019). The inner clock-Blue light sets the human rhythm. J. Biophotonics.

[12] Mosendane T., Mosendane T., Raal F.J. (2008). Shift work and its effects on the cardiovascular system. Cardiovasc. J. Afr..

[13] Wadei H.M., Textor S.C. (2012). The role of the kidney in regulating arterial blood pressure. Nat. Rev. Nephrol..

[14] Johnston J.G., Pollock D.M. (2018). Circadian regulation of renal function. Free Radic. Biol. Med..

[15] Zuther P., Gorbey S., Lemmer B. (2009). Chronos-Fit 1.06. http://chronos-fit.sharewarejunction.com.

[16] Bespyatykh A.Y., Brodskiy V.Y., Burlakova O.V., Golichenkov V.A., Voznesenskaya L.A., Kolesnikov D.B., Molchanov A.Y., Rapoport S.I. (2009). Melatonin: Theory and practice.

[17] Levels of Interleukins and Melatonin in Patients with Acute Coronary Syndrome. https://www.researchgate.net/publication/276311849_LEVELS_OF_INTERLEUKINS_AND_MELATONIN_IN_PATIENTS_WITH_ACUTE_CORONARY_SYNDROME.

[18] Abeysuriya R.G., Lockley S.W., Robinson P.A., Postnova S. (2018). A unified model of melatonin, 6-sulfatoxymelatonin, and sleep dynamics. J. Pineal Res..

[19] Amaral F., Cipolla-Neto J. (2018). A brief review about melatonin, a pineal hormone. Arch. Endocrinol. Metab..

[20] Mistry P., Duong A., Kirshenbaum L., Martino T.A. (2017). Cardiac Clocks and Preclinical Translation. Heart Fail. Clin..

[21] Douma L.G., Gumz M.L. (2018). Circadian clock-mediated regulation of blood pressure. Free Radic. Biol. Med..

[22] Stern M., Broja M., Sansone R., Gröne M., Skene S.S., Liebmann J., Suschek C.V., Born M., Kelm M., Heiss C. (2018). Blue light exposure decreases systolic blood pressure, arterial stiffness, and improves endothelial function in humans. Eur. J. Prev. Cardiol..

[23] Scheer F.A., van Doornen L.J., Buijs R.M. (1999). Light and diurnal cycle affect human heart rate: Possible role for the circadian pacemaker. J. Biol. Rhythm..

[24] Scheer F.A., Van Doornen L.J., Buijs R.M. (2004). Light and diurnal cycle affect autonomic cardiac balance in human; possible role for the biological clock. Auton. Neurosci..

[25] Chellappa S.L., Lasauskaite R., Cajochen C. (2017). In a Heartbeat: Light and Cardiovascular Physiology. Front. Neurol..

[26] Cajochen C., Münch M., Kobialka S., Kräuchi K., Steiner R., Oelhafen P., Orgül S., Wirz-Justice A. (2005). High sensitivity of human melatonin, alertness, thermoregulation, and heart rate to short wavelength light. J. Clin. Endocrinol. Metab..

[27] Modi P., Jha K., Kumar Y., Kumar T., Singh R., Mishra A. (2019). The effect of short-term exposure to red and blue light on the autonomic tone of the individuals with newly diagnosed essential hypertension. J. Family Med. Prim. Care.

[28] Yuda E., Ogasawara H., Yoshida Y., Hayano J. (2016). Suppression of vagal cardiac modulation by blue light in healthy subjects. J. Physiol. Anthropol..

[29] Schäfer A., Kratky K.W. (2006). The effect of colored illumination on heart rate variability. Forsch. Komplementmed..

[30] Blagonravov M.L., Medvedeva E.V., Bryk A.A., Goryachev V.A., Rabinovich A.E., Letoshneva A.S., Demurov E.A. (2018). 24-Hour Profile of Blood Pressure, Heart Rate, Excretion of Electrolytes, and Locomotor Activity in Wistar–Kyoto and SHR Rats Under Conditions of Free-Run Rhythm. Bull. Exp. Biol. Med..

[31] Blagonravov M.L., Bryk A.A., Medvedeva E.V., Goryachev V.A., Chibisov S.M., Kurlaeva A.O., Agafonov E.D. (2019). Structure of Rhythms of Blood Pressure, Heart Rate, Excretion of Electrolytes, and Secretion of Melatonin in Normotensive and Spontaneously Hypertensive Rats Maintained under Conditions of Prolonged Daylight Duration. Bull. Exp. Biol. Med..

[32] Cambar J., Lemoigne F., Toussaint C., Dost C. (1978). Nycthemeral variations of blood and urine urea, creatinine and total proteins in rats. Comptes Rendus Seances Soc. Biol. Fil..

[33] Kamperis K., Hagstroem S., Radvanska E., Rittig S., Djurhuus J.C. (2010). Excess diuresis and natriuresis during acute sleep deprivation in healthy adults. Am. J. Physiol. Renal Physiol..

[34] Crislip G.R., Masten S.H., Gumz M.L. (2018). Recent advances in understanding the circadian clock in renal physiology. Curr. Opin. Physiol..

[35] Bryukhanov V.M., Zvereva A.J. (2010). The kidney role in regulation of circade rithms of the organism. Nephrology.

[36] Stoynev A.G., Ikonomov O.C. (1983). Effect of constant light and darkness on the circadian rhythms in rats: I. Food and water intake, urine output and electrolyte excretion. Acta Physiol. Pharmacol. Bulg..

[37] Mistlberger R.E., Rechtschaffen A. (1985). Periodic water availability is not a potent zeitgeber for entrainment of circadian locomotor rhythms in rats. Physiol. Behav..

[38] Semenenko S.B., Tkachuk S.S., Tkachuk O.V., Karateeva S.Y., Antsupova V.V. (2016). Specific features of chronorhythmologic changes of the ion-regulating function of the kidneys un- der the hypofunction of the pineal gland. Fiziolohichnyi Zhurnal.

[39] Olgaard K., Madsen S., Roosen J., Hammer M. (1977). Circadian rhythm of plasma aldosterone and plasma renin activity in steroid and non-steroid treated kidney transplanted patients. Scand. J. Clin. Lab. Invest..

[40] Doi M., Takahashi Y., Komatsu R., Yamazaki F., Yamada H., Haraguchi S., Emoto N., Okuno Y., Tsujimoto G., Kanematsu A. (2010). Salt-sensitive hypertension in circadian clock-deficient Cry-null mice involves dysregulated adrenal Hsd3b6. Nat. Med..

[41] Firsov D., Bonny O. (2010). Circadian regulation of renal function. Kidney Int..

[42] Ruiz-Hurtado G., Ruilope L.M. (2018). Microvascular injury and the kidney in hypertension. Hipertens. Riesgo Vasc..

[43] Ruilope L.M., Campo C., Lahera V. (1993). The kidney and arterial hypertension. Drugs.

[44] Fang Z., Carlson S.H., Peng N., Wyss J.M. (2000). Circadian rhythm of plasma sodium is disrupted in spontaneously hypertensive rats fed a high-NaCl diet. Am. J. Physiol. Regul. Integr. Comp. Physiol..

[45] Budnevsky A.V., Ovsyannikov E.S., Rezova N.V., Shkatova Y.S. (2017). Melatonin and hypertension: A possible role in combination therapy. Ter. Arkhiv.

[46] Blagonravov M.L., Medvedeva E.V., Bryk A.A., Goryachev V.A., Azova M.M., Velichko E.V. (2017). Specific Features of Electrolyte Excretion at the Early Stages of Arterial Hypertension in SHR Rats. Bull. Exp. Biol. Med..

[47] Bauer M., Glenn T., Monteith S., Gottlieb J.F., Ritter P.S., Geddes J., Whybrow P.C. (2018). The potential influence of LED lighting on mental illness. World J. Biol. Psychiatry.

[48] Cho Y., Ryu S.H., Lee B.R., Kim K.H., Lee E., Choi J. (2015). Effects of artificial light at night on human health: A literature review of observational and experimental studies applied to exposure assessment. Chronobiol. Int..

[49] Touitou Y., Reinberg A., Touitou D. (2017). Association between light at night, melatonin secretion, sleep deprivation, and the internal clock: Health impacts and mechanisms of circadian disruption. Life Sci..

[50] Shanahan T.L., Zeitzer J.M., Czeisler C.A. (1997). Resetting the melatonin rhythm with light in humans. J. Biol. Rhythm..

[51] Prayag A.S., Münch M., Aeschbach D., Chellappa S.L., Gronfier C. (2019). Light Modulation of Human Clocks, Wake, and Sleep. Clocks Sleep.

[52] Wright H.R., Lack L.C. (2001). Effect of light wavelength on suppression and phase delay of the melatonin rhythm. Chronobiol. Int..

[53] Wright H.R., Lack L.C., Kennaway D.J. (2004). Differential effects of light wavelength in phase advancing the melatonin rhythm. J. Pineal Res..

[54] Lockley S.W., Brainard G.C., Czeisler C.A. (2003). High sensitivity of the human circadian melatonin rhythm to resetting by short wavelength light. J. Clin. Endocrinol. Metab..

[55] Arendt J., Skene D.J. (2005). Melatonin as a chronobiotic. Sleep Med. Rev..

